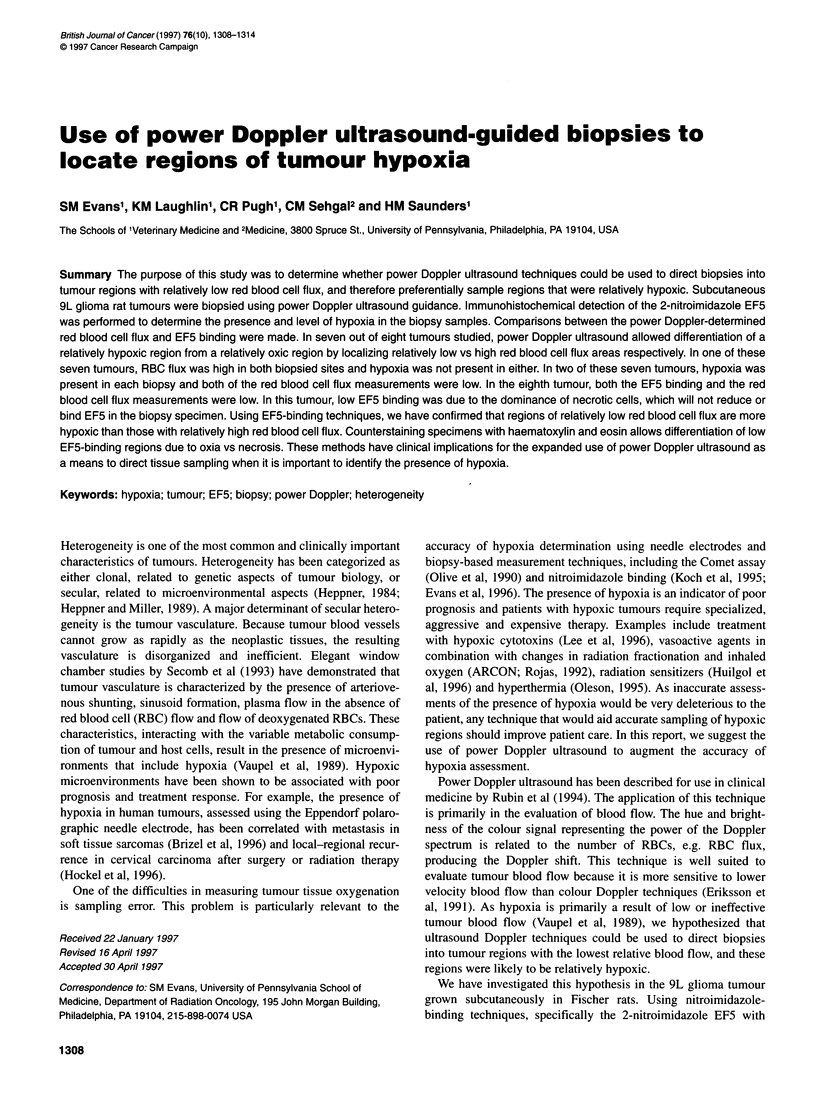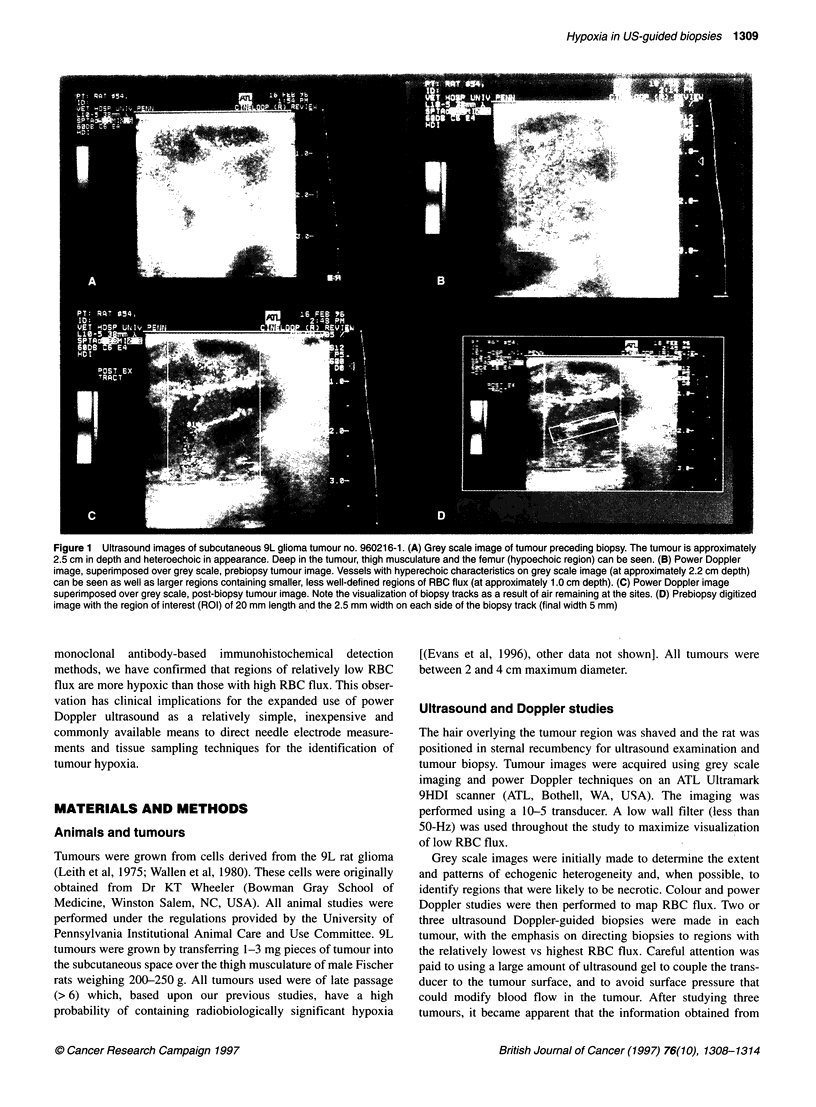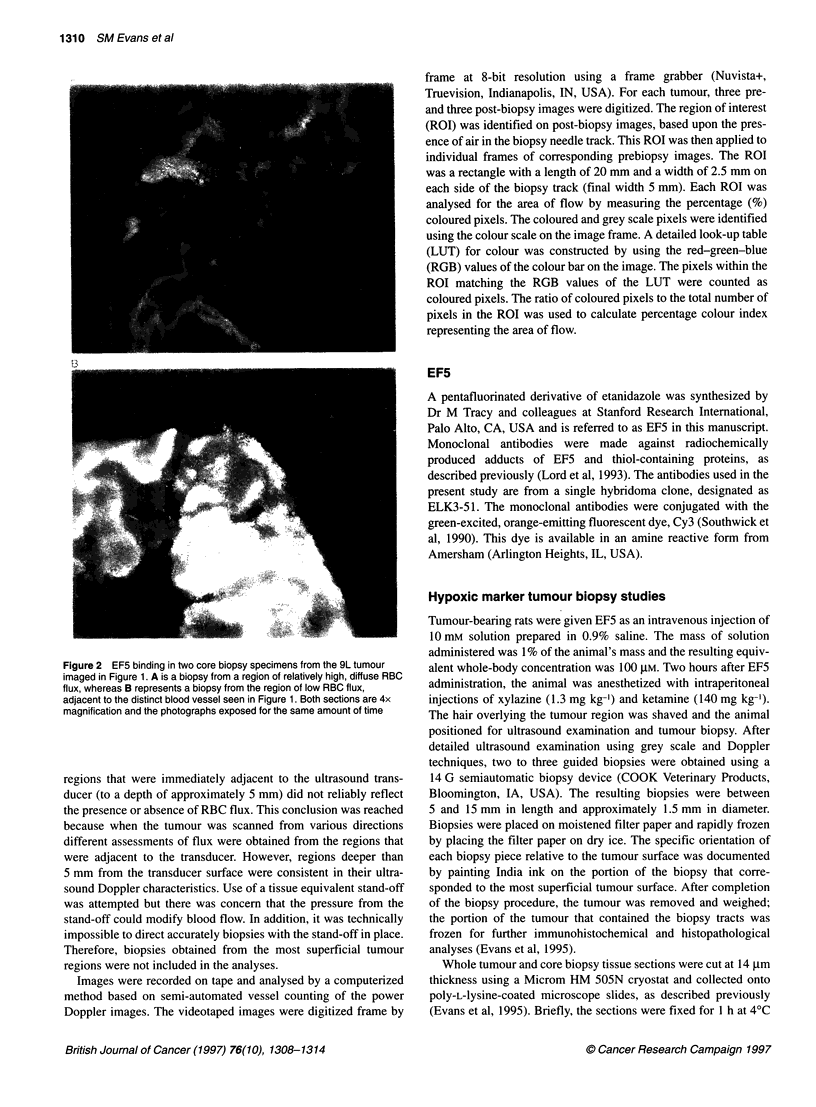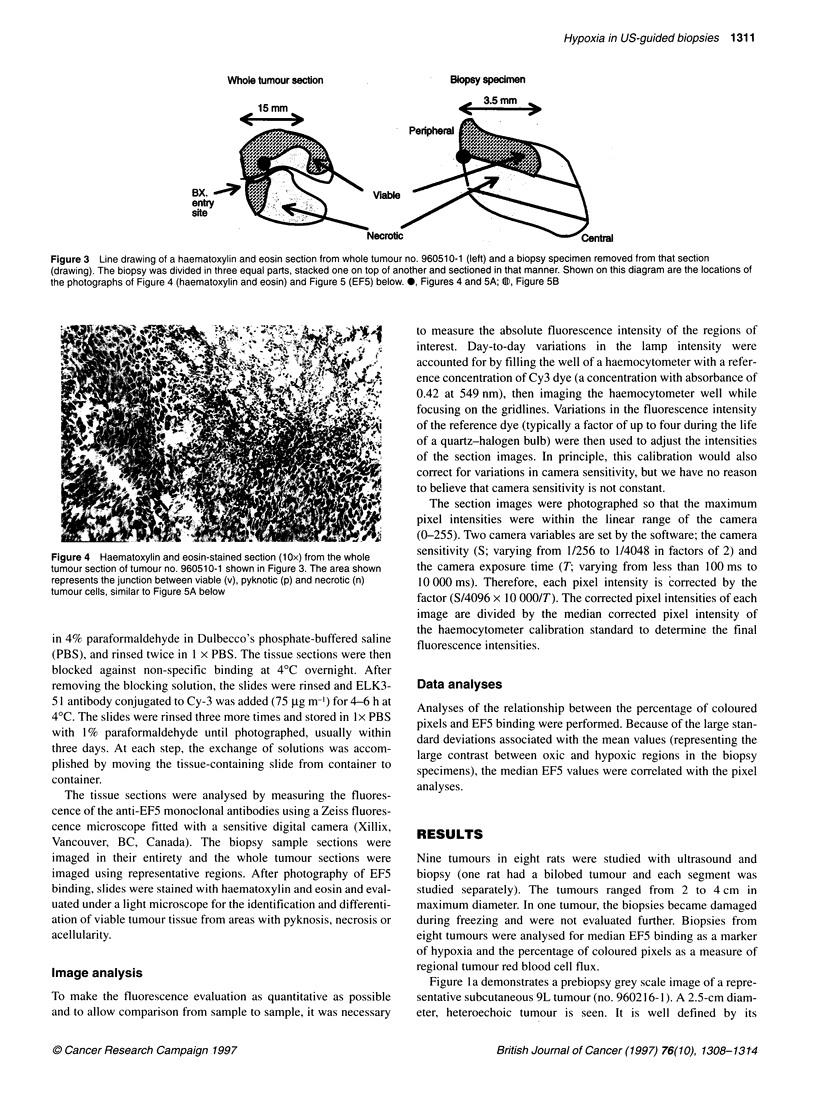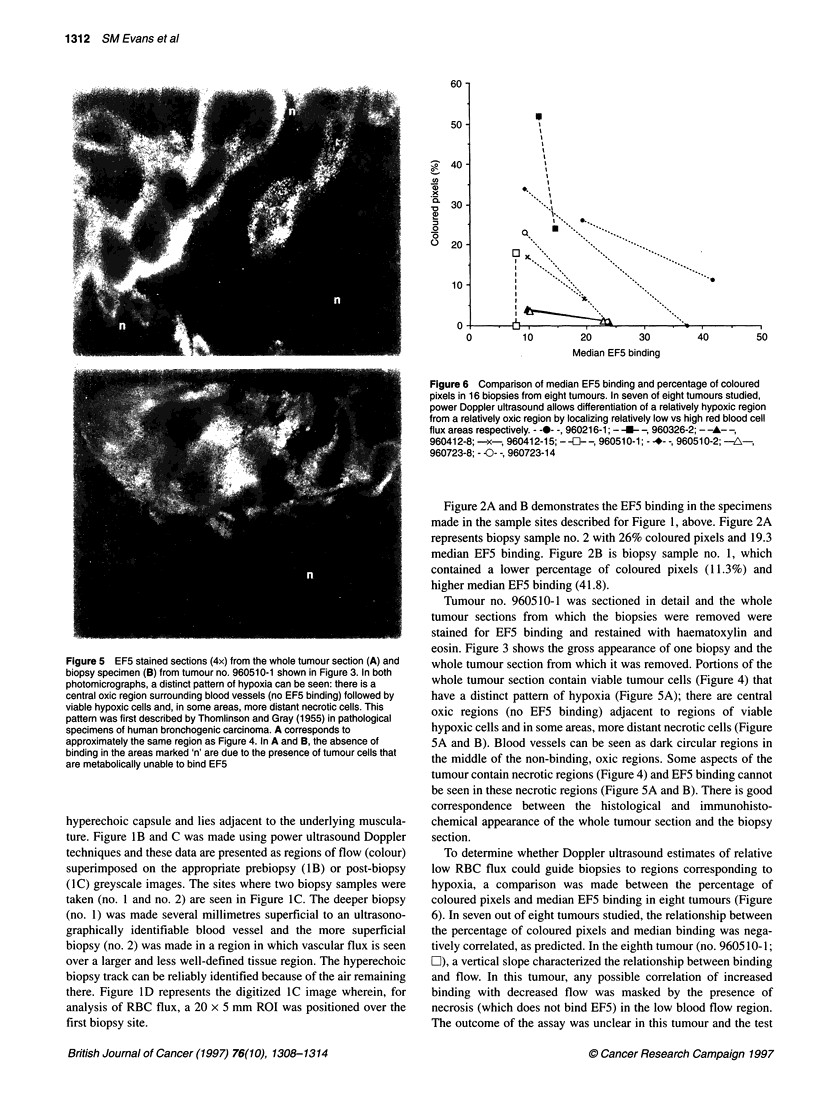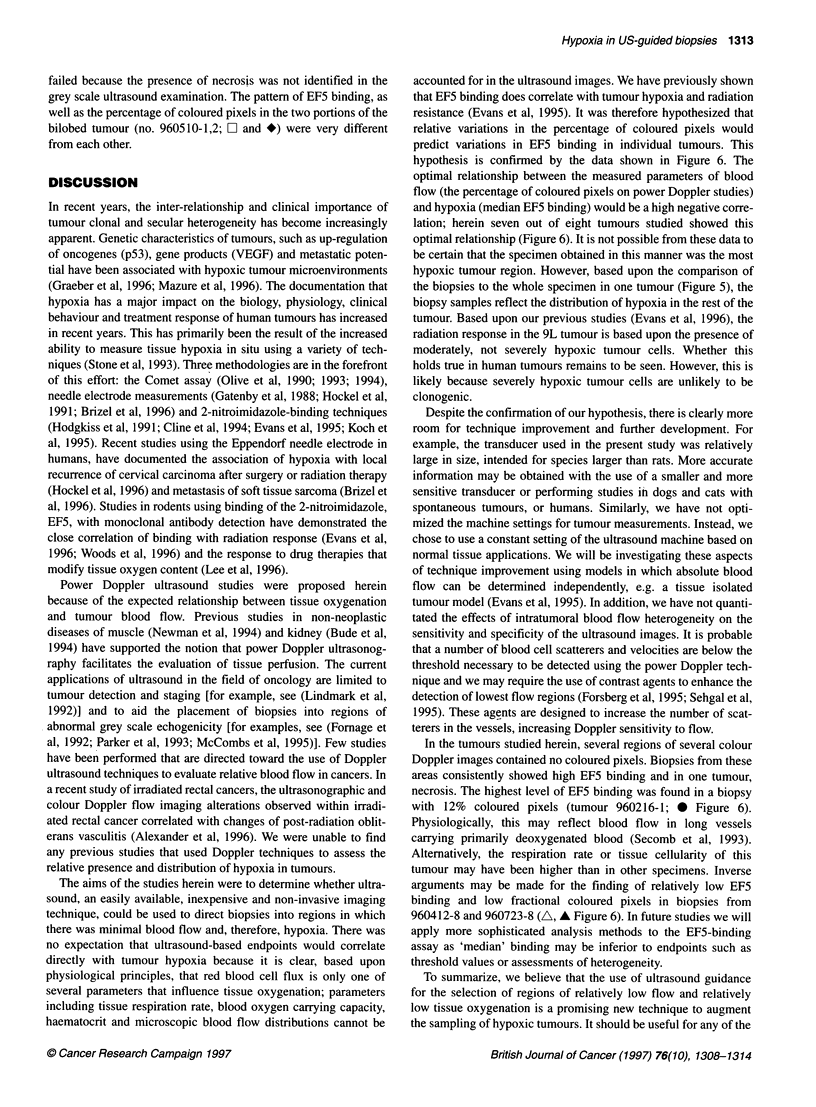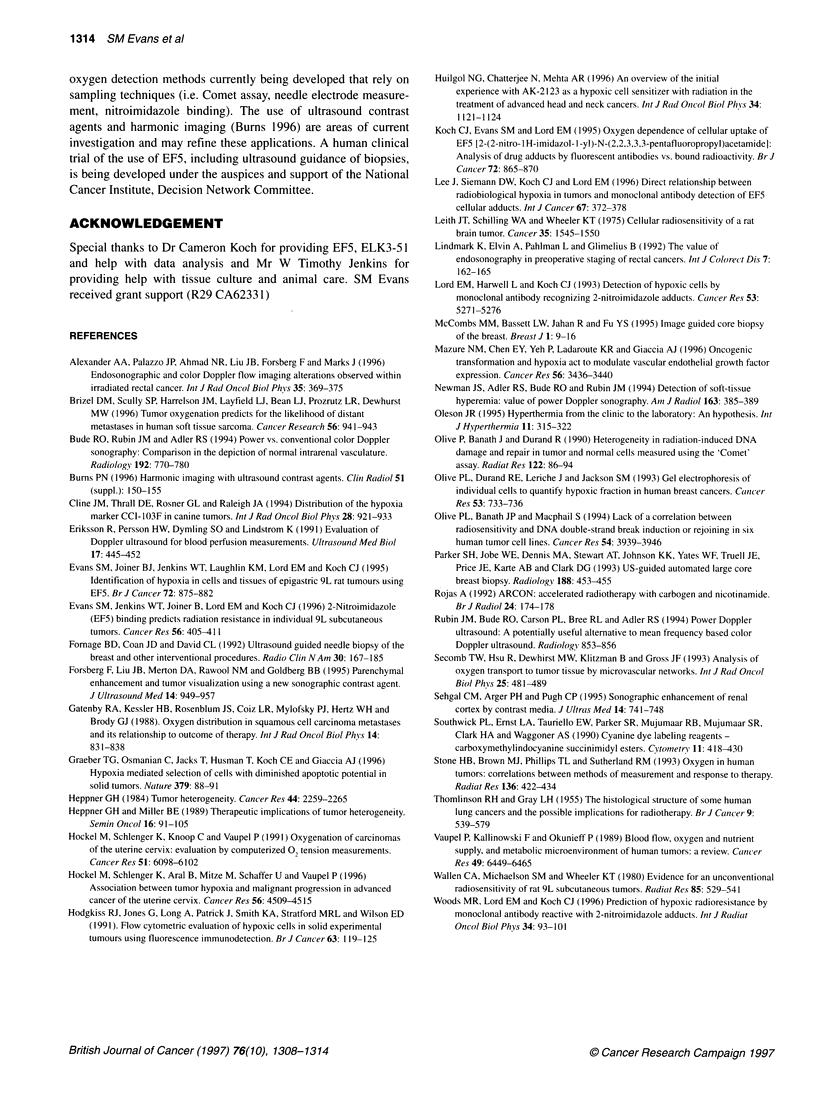# Use of power Doppler ultrasound-guided biopsies to locate regions of tumour hypoxia.

**DOI:** 10.1038/bjc.1997.553

**Published:** 1997

**Authors:** S. M. Evans, K. M. Laughlin, C. R. Pugh, C. M. Sehgal, H. M. Saunders

**Affiliations:** The School of Veterinary Medicine, University of Pennsylvania, Philadelphia 19104, USA.

## Abstract

**Images:**


British Journal of Cancer (1997) 76(10), 1308–1314
© 1997 Cancer Research Campaign

### Use of power Doppler ultrasound-guided biopsies to locate regions of tumour hypoxia

**SM Evans[1], KM Laughlin[1], CR Pugh[1], CM Sehgal[2] and HM Saunders[1]**

The Schools of [1]Veterinary Medicine and [2]Medicine, 3800 Spruce St., University of Pennsylvania, Philadelphia, PA 19104, USA

**Summary** The purpose of this study was to determine whether power Doppler ultrasound techniques could be used to direct biopsies into tumour regions with relatively low red blood cell flux, and therefore preferentially sample regions that were relatively hypoxic. Subcutaneous 9L glioma rat tumours were biopsied using power Doppler ultrasound guidance. Immunohistochemical detection of the 2-nitroimidazole EF5 was performed to determine the presence and level of hypoxia in the biopsy samples. Comparisons between the power Doppler-determined red blood cell flux and EF5 binding were made. In seven out of eight tumours studied, power Doppler ultrasound allowed differentiation of a relatively hypoxic region from a relatively oxic region by localizing relatively low vs high red blood cell flux areas respectively. In one of these seven tumours, RBC flux was high in both biopsied sites and hypoxia was not present in either. In two of these seven tumours, hypoxia was present in each biopsy and both of the red blood cell flux measurements were low. In the eighth tumour, both the EF5 binding and the red blood cell flux measurements were low. In this tumour, low EF5 binding was due to the dominance of necrotic cells, which will not reduce or bind EF5 in the biopsy specimen. Using EF5-binding techniques, we have confirmed that regions of relatively low red blood cell flux are more hypoxic than those with relatively high red blood cell flux. Counterstaining specimens with haematoxylin and eosin allows differentiation of low EF5-binding regions due to oxia vs necrosis. These methods have clinical implications for the expanded use of power Doppler ultrasound as a means to direct tissue sampling when it is important to identify the presence of hypoxia.

**Keywords:** hypoxia; tumour; EF5; biopsy; power Doppler; heterogeneity

Heterogeneity is one of the most common and clinically important characteristics of tumours. Heterogeneity has been categorized as either clonal, related to genetic aspects of tumour biology, or secular, related to microenvironmental aspects (Heppner, 1984; Heppner and Miller, 1989). A major determinant of secular heterogeneity is the tumour vasculature. Because tumour blood vessels cannot grow as rapidly as the neoplastic tissues, the resulting vasculature is disorganized and inefficient. Elegant window chamber studies by Secomb et al (1993) have demonstrated that tumour vasculature is characterized by the presence of arteriovenous shunting, sinusoid formation, plasma flow in the absence of red blood cell (RBC) flow and flow of deoxygenated RBCs. These characteristics, interacting with the variable metabolic consumption of tumour and host cells, result in the presence of microenvironments that include hypoxia (Vaupel et al, 1989). Hypoxic microenvironments have been shown to be associated with poor prognosis and treatment response. For example, the presence of hypoxia in human tumours, assessed using the Eppendorf polarographic needle electrode, has been correlated with metastasis in soft tissue sarcomas (Brizel et al, 1996) and local–regional recurrence in cervical carcinoma after surgery or radiation therapy (Hockel et al, 1996).

One of the difficulties in measuring tumour tissue oxygenation is sampling error. This problem is particularly relevant to the accuracy of hypoxia determination using needle electrodes and biopsy-based measurement techniques, including the Comet assay (Olive et al, 1990) and nitroimidazole binding (Koch et al, 1995; Evans et al, 1996). The presence of hypoxia is an indicator of poor prognosis and patients with hypoxic tumours require specialized, aggressive and expensive therapy. Examples include treatment with hypoxic cytotoxins (Lee et al, 1996), vasoactive agents in combination with changes in radiation fractionation and inhaled oxygen (ARCON; Rojas, 1992), radiation sensitizers (Huilgol et al, 1996) and hyperthermia (Oleson, 1995). As inaccurate assessments of the presence of hypoxia would be very deleterious to the patient, any technique that would aid accurate sampling of hypoxic regions should improve patient care. In this report, we suggest the use of power Doppler ultrasound to augment the accuracy of hypoxia assessment.

Power Doppler ultrasound has been described for use in clinical medicine by Rubin et al (1994). The application of this technique is primarily in the evaluation of blood flow. The hue and brightness of the colour signal representing the power of the Doppler spectrum is related to the number of RBCs, e.g. RBC flux, producing the Doppler shift. This technique is well suited to evaluate tumour blood flow because it is more sensitive to lower velocity blood flow than colour Doppler techniques (Eriksson et al, 1991). As hypoxia is primarily a result of low or ineffective tumour blood flow (Vaupel et al, 1989), we hypothesized that ultrasound Doppler techniques could be used to direct biopsies into tumour regions with the lowest relative blood flow, and these regions were likely to be relatively hypoxic.

We have investigated this hypothesis in the 9L glioma tumour grown subcutaneously in Fischer rats. Using nitroimidazole-binding techniques, specifically the 2-nitroimidazole EF5 with

*Received 22 January 1997*
*Revised 16 April 1997*
*Accepted 30 April 1997*

*Correspondence to:* SM Evans, University of Pennsylvania School of Medicine, Department of Radiation Oncology, 195 John Morgan Building, Philadelphia, PA 19104, 215-898-0074 USA

**Figure 1** Ultrasound images of subcutaneous 9L glioma tumour no. 960216-1. (**A**) Grey scale image of tumour preceding biopsy. The tumour is approximately 2.5 cm in depth and heteroechoic in appearance. Deep in the tumour, thigh musculature and the femur (hypoechoic region) can be seen. (**B**) Power Doppler image, superimposed over grey scale, prebiopsy tumour image. Vessels with hyperechoic characteristics on grey scale image (at approximately 2.2 cm depth) can be seen as well as larger regions containing smaller, less well-defined regions of RBC flux (at approximately 1.0 cm depth). (**C**) Power Doppler image superimposed over grey scale, post-biopsy tumour image. Note the visualization of biopsy tracks as a result of air remaining at the sites. (**D**) Prebiopsy digitized image with the region of interest (ROI) of 20 mm length and the 2.5 mm width on each side of the biopsy track (final width 5 mm)

monoclonal antibody-based immunohistochemical detection methods, we have confirmed that regions of relatively low RBC flux are more hypoxic than those with high RBC flux. This observation has clinical implications for the expanded use of power Doppler ultrasound as a relatively simple, inexpensive and commonly available means to direct needle electrode measurements and tissue sampling techniques for the identification of tumour hypoxia.

### MATERIALS AND METHODS

### Animals and tumours

Tumours were grown from cells derived from the 9L rat glioma (Leith et al, 1975; Wallen et al, 1980). These cells were originally obtained from Dr KT Wheeler (Bowman Gray School of Medicine, Winston Salem, NC, USA). All animal studies were performed under the regulations provided by the University of Pennsylvania Institutional Animal Care and Use Committee. 9L tumours were grown by transferring 1–3 mg pieces of tumour into the subcutaneous space over the thigh musculature of male Fischer rats weighing 200–250 g. All tumours used were of late passage (> 6) which, based upon our previous studies, have a high probability of containing radiobiologically significant hypoxia [(Evans et al, 1996), other data not shown]. All tumours were between 2 and 4 cm maximum diameter.

### Ultrasound and Doppler studies

The hair overlying the tumour region was shaved and the rat was positioned in sternal recumbency for ultrasound examination and tumour biopsy. Tumour images were acquired using grey scale imaging and power Doppler techniques on an ATL Ultramark 9HDI scanner (ATL, Bothell, WA, USA). The imaging was performed using a 10–5 transducer. A low wall filter (less than 50-Hz) was used throughout the study to maximize visualization of low RBC flux.

Grey scale images were initially made to determine the extent and patterns of echogenic heterogeneity and, when possible, to identify regions that were likely to be necrotic. Colour and power Doppler studies were then performed to map RBC flux. Two or three ultrasound Doppler-guided biopsies were made in each tumour, with the emphasis on directing biopsies to regions with the relatively lowest vs highest RBC flux. Careful attention was paid to using a large amount of ultrasound gel to couple the transducer to the tumour surface, and to avoid surface pressure that could modify blood flow in the tumour. After studying three tumours, it became apparent that the information obtained from

*© Cancer Research Campaign 1997* 

**Figure 2** EF5 binding in two core biopsy specimens from the 9L tumour imaged in Figure 1. **A** is a biopsy from a region of relatively high, diffuse RBC flux, whereas **B** represents a biopsy from the region of low RBC flux, adjacent to the distinct blood vessel seen in Figure 1. Both sections are 4× magnification and the photographs exposed for the same amount of time regions that were immediately adjacent to the ultrasound transducer (to a depth of approximately 5 mm) did not reliably reflect the presence or absence of RBC flux. This conclusion was reached because when the tumour was scanned from various directions different assessments of flux were obtained from the regions that were adjacent to the transducer. However, regions deeper than 5 mm from the transducer surface were consistent in their ultrasound Doppler characteristics. Use of a tissue equivalent stand-off was attempted but there was concern that the pressure from the stand-off could modify blood flow. In addition, it was technically impossible to direct accurately biopsies with the stand-off in place. Therefore, biopsies obtained from the most superficial tumour regions were not included in the analyses.

Images were recorded on tape and analysed by a computerized method based on semi-automated vessel counting of the power Doppler images. The videotaped images were digitized frame by frame at 8-bit resolution using a frame grabber (Nuvista+, Truevision, Indianapolis, IN, USA). For each tumour, three pre- and three post-biopsy images were digitized. The region of interest (ROI) was identified on post-biopsy images, based upon the presence of air in the biopsy needle track. This ROI was then applied to individual frames of corresponding prebiopsy images. The ROI was a rectangle with a length of 20 mm and a width of 2.5 mm on each side of the biopsy track (final width 5 mm). Each ROI was analysed for the area of flow by measuring the percentage (%) coloured pixels. The coloured and grey scale pixels were identified using the colour scale on the image frame. A detailed look-up table (LUT) for colour was constructed by using the red–green–blue (RGB) values of the colour bar on the image. The pixels within the ROI matching the RGB values of the LUT were counted as coloured pixels. The ratio of coloured pixels to the total number of pixels in the ROI was used to calculate percentage colour index representing the area of flow.

### EF5

A pentafluorinated derivative of etanidazole was synthesized by Dr M Tracy and colleagues at Stanford Research International, Palo Alto, CA, USA and is referred to as EF5 in this manuscript. Monoclonal antibodies were made against radiochemically produced adducts of EF5 and thiol-containing proteins, as described previously (Lord et al, 1993). The antibodies used in the present study are from a single hybridoma clone, designated as ELK3-51. The monoclonal antibodies were conjugated with the green-excited, orange-emitting fluorescent dye, Cy3 (Southwick et al, 1990). This dye is available in an amine reactive form from Amersham (Arlington Heights, IL, USA).

### Hypoxic marker tumour biopsy studies

Tumour-bearing rats were given EF5 as an intravenous injection of 10 mM solution prepared in 0.9% saline. The mass of solution administered was 1% of the animal's mass and the resulting equivalent whole-body concentration was 100 μM. Two hours after EF5 administration, the animal was anesthetized with intraperitoneal injections of xylazine (1.3 mg $kg^{-1}$) and ketamine (140 mg $kg^{-1}$). The hair overlying the tumour region was shaved and the animal positioned for ultrasound examination and tumour biopsy. After detailed ultrasound examination using grey scale and Doppler techniques, two to three guided biopsies were obtained using a 14 G semiautomatic biopsy device (COOK Veterinary Products, Bloomington, IA, USA). The resulting biopsies were between 5 and 15 mm in length and approximately 1.5 mm in diameter. Biopsies were placed on moistened filter paper and rapidly frozen by placing the filter paper on dry ice. The specific orientation of each biopsy piece relative to the tumour surface was documented by painting India ink on the portion of the biopsy that corresponded to the most superficial tumour surface. After completion of the biopsy procedure, the tumour was removed and weighed; the portion of the tumour that contained the biopsy tracts was frozen for further immunohistochemical and histopathological analyses (Evans et al, 1995).

Whole tumour and core biopsy tissue sections were cut at 14 μm thickness using a Microm HM 505N cryostat and collected onto poly-L-lysine-coated microscope slides, as described previously (Evans et al, 1995). Briefly, the sections were fixed for 1 h at 4°C

© Cancer Research Campaign 1997

**Figure 3** Line drawing of a haematoxylin and eosin section from whole tumour no. 960510-1 (left) and a biopsy specimen removed from that section (drawing). The biopsy was divided in three equal parts, stacked one on top of another and sectioned in that manner. Shown on this diagram are the locations of the photographs of Figure 4 (haematoxylin and eosin) and Figure 5 (EF5) below. ●, Figures 4 and 5A; ◍, Figure 5B

**Figure 4** Haematoxylin and eosin-stained section (10×) from the whole tumour section of tumour no. 960510-1 shown in Figure 3. The area shown represents the junction between viable (v), pyknotic (p) and necrotic (n) tumour cells, similar to Figure 5A below in 4% paraformaldehyde in Dulbecco's phosphate-buffered saline (PBS), and rinsed twice in 1 × PBS. The tissue sections were then blocked against non-specific binding at 4°C overnight. After removing the blocking solution, the slides were rinsed and ELK3-51 antibody conjugated to Cy-3 was added (75 µg $m^{-1}$) for 4–6 h at 4°C. The slides were rinsed three more times and stored in 1× PBS with 1% paraformaldehyde until photographed, usually within three days. At each step, the exchange of solutions was accomplished by moving the tissue-containing slide from container to container.

The tissue sections were analysed by measuring the fluorescence of the anti-EF5 monoclonal antibodies using a Zeiss fluorescence microscope fitted with a sensitive digital camera (Xillix, Vancouver, BC, Canada). The biopsy sample sections were imaged in their entirety and the whole tumour sections were imaged using representative regions. After photography of EF5 binding, slides were stained with haematoxylin and eosin and evaluated under a light microscope for the identification and differentiation of viable tumour tissue from areas with pyknosis, necrosis or acellularity.

### Image analysis

To make the fluorescence evaluation as quantitative as possible and to allow comparison from sample to sample, it was necessary to measure the absolute fluorescence intensity of the regions of interest. Day-to-day variations in the lamp intensity were accounted for by filling the well of a haemocytometer with a reference concentration of Cy3 dye (a concentration with absorbance of 0.42 at 549 nm), then imaging the haemocytometer well while focusing on the gridlines. Variations in the fluorescence intensity of the reference dye (typically a factor of up to four during the life of a quartz–halogen bulb) were then used to adjust the intensities of the section images. In principle, this calibration would also correct for variations in camera sensitivity, but we have no reason to believe that camera sensitivity is not constant.

The section images were photographed so that the maximum pixel intensities were within the linear range of the camera (0–255). Two camera variables are set by the software; the camera sensitivity (S; varying from 1/256 to 1/4048 in factors of 2) and the camera exposure time (*T*; varying from less than 100 ms to 10 000 ms). Therefore, each pixel intensity is corrected by the factor (S/4096 × 10 000/*T*). The corrected pixel intensities of each image are divided by the median corrected pixel intensity of the haemocytometer calibration standard to determine the final fluorescence intensities.

### Data analyses

Analyses of the relationship between the percentage of coloured pixels and EF5 binding were performed. Because of the large standard deviations associated with the mean values (representing the large contrast between oxic and hypoxic regions in the biopsy specimens), the median EF5 values were correlated with the pixel analyses.

### RESULTS

Nine tumours in eight rats were studied with ultrasound and biopsy (one rat had a bilobed tumour and each segment was studied separately). The tumours ranged from 2 to 4 cm in maximum diameter. In one tumour, the biopsies became damaged during freezing and were not evaluated further. Biopsies from eight tumours were analysed for median EF5 binding as a marker of hypoxia and the percentage of coloured pixels as a measure of regional tumour red blood cell flux.

Figure 1a demonstrates a prebiopsy grey scale image of a representative subcutaneous 9L tumour (no. 960216-1). A 2.5-cm diameter, heteroechoic tumour is seen. It is well defined by its

**Figure 5** EF5 stained sections (4×) from the whole tumour section (**A**) and biopsy specimen (**B**) from tumour no. 960510-1 shown in Figure 3. In both photomicrographs, a distinct pattern of hypoxia can be seen: there is a central oxic region surrounding blood vessels (no EF5 binding) followed by viable hypoxic cells and, in some areas, more distant necrotic cells. This pattern was first described by Thomlinson and Gray (1955) in pathological specimens of human bronchogenic carcinoma. **A** corresponds to approximately the same region as Figure 4. In **A** and **B**, the absence of binding in the areas marked 'n' are due to the presence of tumour cells that are metabolically unable to bind EF5

**Figure 6** Comparison of median EF5 binding and percentage of coloured pixels in 16 biopsies from eight tumours. In seven of eight tumours studied, power Doppler ultrasound allows differentiation of a relatively hypoxic region from a relatively oxic region by localizing relatively low vs high red blood cell flux areas respectively. - -●- -, 960216-1; – –■– –, 960326-2; – –▲– –, 960412-8; —×—, 960412-15; – –□– –, 960510-1; - -◆- -, 960510-2; —△—, 960723-8; - -○- -, 960723-14

hyperechoic capsule and lies adjacent to the underlying musculature. Figure 1B and C was made using power ultrasound Doppler techniques and these data are presented as regions of flow (colour) superimposed on the appropriate prebiopsy (1B) or post-biopsy (1C) greyscale images. The sites where two biopsy samples were taken (no. 1 and no. 2) are seen in Figure 1C. The deeper biopsy (no. 1) was made several millimetres superficial to an ultrasonographically identifiable blood vessel and the more superficial biopsy (no. 2) was made in a region in which vascular flux is seen over a larger and less well-defined tissue region. The hyperechoic biopsy track can be reliably identified because of the air remaining there. Figure 1D represents the digitized 1C image wherein, for analysis of RBC flux, a 20 × 5 mm ROI was positioned over the first biopsy site.

Figure 2A and B demonstrates the EF5 binding in the specimens made in the sample sites described for Figure 1, above. Figure 2A represents biopsy sample no. 2 with 26% coloured pixels and 19.3 median EF5 binding. Figure 2B is biopsy sample no. 1, which contained a lower percentage of coloured pixels (11.3%) and higher median EF5 binding (41.8).

Tumour no. 960510-1 was sectioned in detail and the whole tumour sections from which the biopsies were removed were stained for EF5 binding and restained with haematoxylin and eosin. Figure 3 shows the gross appearance of one biopsy and the whole tumour section from which it was removed. Portions of the whole tumour section contain viable tumour cells (Figure 4) that have a distinct pattern of hypoxia (Figure 5A); there are central oxic regions (no EF5 binding) adjacent to regions of viable hypoxic cells and in some areas, more distant necrotic cells (Figure 5A and B). Blood vessels can be seen as dark circular regions in the middle of the non-binding, oxic regions. Some aspects of the tumour contain necrotic regions (Figure 4) and EF5 binding cannot be seen in these necrotic regions (Figure 5A and B). There is good correspondence between the histological and immunohistochemical appearance of the whole tumour section and the biopsy section.

To determine whether Doppler ultrasound estimates of relative low RBC flux could guide biopsies to regions corresponding to hypoxia, a comparison was made between the percentage of coloured pixels and median EF5 binding in eight tumours (Figure 6). In seven out of eight tumours studied, the relationship between the percentage of coloured pixels and median binding was negatively correlated, as predicted. In the eighth tumour (no. 960510-1; □), a vertical slope characterized the relationship between binding and flow. In this tumour, any possible correlation of increased binding with decreased flow was masked by the presence of necrosis (which does not bind EF5) in the low blood flow region. The outcome of the assay was unclear in this tumour and the test

*© Cancer Research Campaign 1997*

failed because the presence of necrosis was not identified in the grey scale ultrasound examination. The pattern of EF5 binding, as well as the percentage of coloured pixels in the two portions of the bilobed tumour (no. 960510-1,2; □ and ◆) were very different from each other.

### DISCUSSION

In recent years, the inter-relationship and clinical importance of tumour clonal and secular heterogeneity has become increasingly apparent. Genetic characteristics of tumours, such as up-regulation of oncogenes (p53), gene products (VEGF) and metastatic potential have been associated with hypoxic tumour microenvironments (Graeber et al, 1996; Mazure et al, 1996). The documentation that hypoxia has a major impact on the biology, physiology, clinical behaviour and treatment response of human tumours has increased in recent years. This has primarily been the result of the increased ability to measure tissue hypoxia in situ using a variety of techniques (Stone et al, 1993). Three methodologies are in the forefront of this effort: the Comet assay (Olive et al, 1990; 1993; 1994), needle electrode measurements (Gatenby et al, 1988; Hockel et al, 1991; Brizel et al, 1996) and 2-nitroimidazole-binding techniques (Hodgkiss et al, 1991; Cline et al, 1994; Evans et al, 1995; Koch et al, 1995). Recent studies using the Eppendorf needle electrode in humans, have documented the association of hypoxia with local recurrence of cervical carcinoma after surgery or radiation therapy (Hockel et al, 1996) and metastasis of soft tissue sarcoma (Brizel et al, 1996). Studies in rodents using binding of the 2-nitroimidazole, EF5, with monoclonal antibody detection have demonstrated the close correlation of binding with radiation response (Evans et al, 1996; Woods et al, 1996) and the response to drug therapies that modify tissue oxygen content (Lee et al, 1996).

Power Doppler ultrasound studies were proposed herein because of the expected relationship between tissue oxygenation and tumour blood flow. Previous studies in non-neoplastic diseases of muscle (Newman et al, 1994) and kidney (Bude et al, 1994) have supported the notion that power Doppler ultrasonography facilitates the evaluation of tissue perfusion. The current applications of ultrasound in the field of oncology are limited to tumour detection and staging [for example, see (Lindmark et al, 1992)] and to aid the placement of biopsies into regions of abnormal grey scale echogenicity [for examples, see (Fornage et al, 1992; Parker et al, 1993; McCombs et al, 1995)]. Few studies have been performed that are directed toward the use of Doppler ultrasound techniques to evaluate relative blood flow in cancers. In a recent study of irradiated rectal cancers, the ultrasonographic and colour Doppler flow imaging alterations observed within irradiated rectal cancer correlated with changes of post-radiation obliterans vasculitis (Alexander et al, 1996). We were unable to find any previous studies that used Doppler techniques to assess the relative presence and distribution of hypoxia in tumours.

The aims of the studies herein were to determine whether ultrasound, an easily available, inexpensive and non-invasive imaging technique, could be used to direct biopsies into regions in which there was minimal blood flow and, therefore, hypoxia. There was no expectation that ultrasound-based endpoints would correlate directly with tumour hypoxia because it is clear, based upon physiological principles, that red blood cell flux is only one of several parameters that influence tissue oxygenation; parameters including tissue respiration rate, blood oxygen carrying capacity, haematocrit and microscopic blood flow distributions cannot be accounted for in the ultrasound images. We have previously shown that EF5 binding does correlate with tumour hypoxia and radiation resistance (Evans et al, 1995). It was therefore hypothesized that relative variations in the percentage of coloured pixels would predict variations in EF5 binding in individual tumours. This hypothesis is confirmed by the data shown in Figure 6. The optimal relationship between the measured parameters of blood flow (the percentage of coloured pixels on power Doppler studies) and hypoxia (median EF5 binding) would be a high negative correlation; herein seven out of eight tumours studied showed this optimal relationship (Figure 6). It is not possible from these data to be certain that the specimen obtained in this manner was the most hypoxic tumour region. However, based upon the comparison of the biopsies to the whole specimen in one tumour (Figure 5), the biopsy samples reflect the distribution of hypoxia in the rest of the tumour. Based upon our previous studies (Evans et al, 1996), the radiation response in the 9L tumour is based upon the presence of moderately, not severely hypoxic tumour cells. Whether this holds true in human tumours remains to be seen. However, this is likely because severely hypoxic tumour cells are unlikely to be clonogenic.

Despite the confirmation of our hypothesis, there is clearly more room for technique improvement and further development. For example, the transducer used in the present study was relatively large in size, intended for species larger than rats. More accurate information may be obtained with the use of a smaller and more sensitive transducer or performing studies in dogs and cats with spontaneous tumours, or humans. Similarly, we have not optimized the machine settings for tumour measurements. Instead, we chose to use a constant setting of the ultrasound machine based on normal tissue applications. We will be investigating these aspects of technique improvement using models in which absolute blood flow can be determined independently, e.g. a tissue isolated tumour model (Evans et al, 1995). In addition, we have not quantitated the effects of intratumoral blood flow heterogeneity on the sensitivity and specificity of the ultrasound images. It is probable that a number of blood cell scatterers and velocities are below the threshold necessary to be detected using the power Doppler technique and we may require the use of contrast agents to enhance the detection of lowest flow regions (Forsberg et al, 1995; Sehgal et al, 1995). These agents are designed to increase the number of scatterers in the vessels, increasing Doppler sensitivity to flow.

In the tumours studied herein, several regions of several colour Doppler images contained no coloured pixels. Biopsies from these areas consistently showed high EF5 binding and in one tumour, necrosis. The highest level of EF5 binding was found in a biopsy with 12% coloured pixels (tumour 960216-1; ● Figure 6). Physiologically, this may reflect blood flow in long vessels carrying primarily deoxygenated blood (Secomb et al, 1993). Alternatively, the respiration rate or tissue cellularity of this tumour may have been higher than in other specimens. Inverse arguments may be made for the finding of relatively low EF5 binding and low fractional coloured pixels in biopsies from 960412-8 and 960723-8 (△, ▲ Figure 6). In future studies we will apply more sophisticated analysis methods to the EF5-binding assay as 'median' binding may be inferior to endpoints such as threshold values or assessments of heterogeneity.

To summarize, we believe that the use of ultrasound guidance for the selection of regions of relatively low flow and relatively low tissue oxygenation is a promising new technique to augment the sampling of hypoxic tumours. It should be useful for any of the

© Cancer Research Campaign 1997

oxygen detection methods currently being developed that rely on sampling techniques (i.e. Comet assay, needle electrode measurement, nitroimidazole binding). The use of ultrasound contrast agents and harmonic imaging (Burns 1996) are areas of current investigation and may refine these applications. A human clinical trial of the use of EF5, including ultrasound guidance of biopsies, is being developed under the auspices and support of the National Cancer Institute, Decision Network Committee.

### ACKNOWLEDGEMENT

Special thanks to Dr Cameron Koch for providing EF5, ELK3-51 and help with data analysis and Mr W Timothy Jenkins for providing help with tissue culture and animal care. SM Evans received grant support (R29 CA62331)

### REFERENCES

Alexander AA, Palazzo JP, Ahmad NR, Liu JB, Forsberg F and Marks J (1996) Endosonographic and color Doppler flow imaging alterations observed within irradiated rectal cancer. *Int J Rad Oncol Biol Phys* **35**: 369–375

Brizel DM, Scully SP, Harrelson JM, Layfield LJ, Bean LJ, Prozrutz LR, Dewhurst MW (1996) Tumor oxygenation predicts for the likelihood of distant metastases in human soft tissue sarcoma. *Cancer Research* **56**: 941–943

Bude RO, Rubin JM and Adler RS (1994) Power vs. conventional color Doppler sonography: Comparison in the depiction of normal intrarenal vasculature. *Radiology* **192**: 770–780

Burns PN (1996) Harmonic imaging with ultrasound contrast agents. *Clin Radiol* **51** (suppl.): 150–155

Cline JM, Thrall DE, Rosner GL and Raleigh JA (1994) Distribution of the hypoxia marker CCI-103F in canine tumors. *Int J Rad Oncol Biol Phys* **28**: 921–933

Eriksson R, Persson HW, Dymling SO and Lindstrom K (1991) Evaluation of Doppler ultrasound for blood perfusion measurements. *Ultrasound Med Biol* **17**: 445–452

Evans SM, Joiner BJ, Jenkins WT, Laughlin KM, Lord EM and Koch CJ (1995) Identification of hypoxia in cells and tissues of epigastric 9L rat tumours using EF5. *Br J Cancer* **72**: 875–882

Evans SM, Jenkins WT, Joiner B, Lord EM and Koch CJ (1996) 2-Nitroimidazole (EF5) binding predicts radiation resistance in individual 9L subcutaneous tumors. *Cancer Res* **56**: 405–411

Fornage BD, Coan JD and David CL (1992) Ultrasound guided needle biopsy of the breast and other interventional procedures. *Radio Clin N Am* **30**: 167–185

Forsberg F, Liu JB, Merton DA, Rawool NM and Goldberg BB (1995) Parenchymal enhancement and tumor visualization using a new sonographic contrast agent. *J Ultrasound Med* **14**: 949–957

Gatenby RA, Kessler HB, Rosenblum JS, Coiz LR, Mylofsky PJ, Hertz WH and Brody GJ (1988). Oxygen distribution in squamous cell carcinoma metastases and its relationship to outcome of therapy. *Int J Rad Oncol Biol Phys* **14**: 831–838

Graeber TG, Osmanian C, Jacks T, Husman T, Koch CE and Giaccia AJ (1996) Hypoxia mediated selection of cells with diminished apoptotic potential in solid tumors. *Nature* **379**: 88–91

Heppner GH (1984) Tumor heterogeneity. *Cancer Res* **44**: 2259–2265

Heppner GH and Miller BE (1989) Therapeutic implications of tumor heterogeneity. *Semin Oncol* **16**: 91–105

Hockel M, Schlenger K, Knoop C and Vaupel P (1991) Oxygenation of carcinomas of the uterine cervix: evaluation by computerized $O_2$ tension measurements. *Cancer Res* **51**: 6098–6102

Hockel M, Schlenger K, Aral B, Mitze M, Schaffer U and Vaupel P (1996) Association between tumor hypoxia and malignant progression in advanced cancer of the uterine cervix. *Cancer Res* **56**: 4509–4515

Hodgkiss RJ, Jones G, Long A, Patrick J, Smith KA, Stratford MRL and Wilson ED (1991). Flow cytometric evaluation of hypoxic cells in solid experimental tumours using fluorescence immunodetection. *Br J Cancer* **63**: 119–125

Huilgol NG, Chatterjee N, Mehta AR (1996) An overview of the initial experience with AK-2123 as a hypoxic cell sensitizer with radiation in the treatment of advanced head and neck cancers. *Int J Rad Oncol Biol Phys* **34**: 1121–1124

Koch CJ, Evans SM and Lord EM (1995) Oxygen dependence of cellular uptake of EF5 [2-(2-nitro-1H-imidazol-1-yl)-N-(2,2,3,3,3-pentafluoropropyl)acetamide]: Analysis of drug adducts by fluorescent antibodies vs. bound radioactivity. *Br J Cancer* **72**: 865–870

Lee J, Siemann DW, Koch CJ and Lord EM (1996) Direct relationship between radiobiological hypoxia in tumors and monoclonal antibody detection of EF5 cellular adducts. *Int J Cancer* **67**: 372–378

Leith JT, Schilling WA and Wheeler KT (1975) Cellular radiosensitivity of a rat brain tumor. *Cancer* **35**: 1545–1550

Lindmark K, Elvin A, Pahlman L and Glimelius B (1992) The value of endosonography in preoperative staging of rectal cancers. *Int J Colorect Dis* **7**: 162–165

Lord EM, Harwell L and Koch CJ (1993) Detection of hypoxic cells by monoclonal antibody recognizing 2-nitroimidazole adducts. *Cancer Res* **53**: 5271–5276

McCombs MM, Bassett LW, Jahan R and Fu YS (1995) Image guided core biopsy of the breast. *Breast J* **1**: 9–16

Mazure NM, Chen EY, Yeh P, Ladaroute KR and Giaccia AJ (1996) Oncogenic transformation and hypoxia act to modulate vascular endothelial growth factor expression. *Cancer Res* **56**: 3436–3440

Newman JS, Adler RS, Bude RO and Rubin JM (1994) Detection of soft-tissue hyperemia: value of power Doppler sonography. *Am J Radiol* **163**: 385–389

Oleson JR (1995) Hyperthermia from the clinic to the laboratory: An hypothesis. *Int J Hyperthermia* **11**: 315–322

Olive P, Banath J and Durand R (1990) Heterogeneity in radiation-induced DNA damage and repair in tumor and normal cells measured using the 'Comet' assay. *Radiat Res* **122**: 86–94

Olive PL, Durand RE, Leriche J and Jackson SM (1993) Gel electrophoresis of individual cells to quantify hypoxic fraction in human breast cancers. *Cancer Res* **53**: 733–736

Olive PL, Banath JP and Macphail S (1994) Lack of a correlation between radiosensitivity and DNA double-strand break induction or rejoining in six human tumor cell lines. *Cancer Res* **54**: 3939–3946

Parker SH, Jobe WE, Dennis MA, Stewart AT, Johnson KK, Yates WF, Truell JE, Price JE, Karte AB and Clark DG (1993) US-guided automated large core breast biopsy. *Radiology* **188**: 453–455

Rojas A (1992) ARCON: accelerated radiotherapy with carbogen and nicotinamide. *Br J Radiol* **24**: 174–178

Rubin JM, Bude RO, Carson PL, Bree RL and Adler RS (1994) Power Doppler ultrasound: A potentially useful alternative to mean frequency based color Doppler ultrasound. *Radiology* 853–856

Secomb TW, Hsu R, Dewhirst MW, Klitzman B and Gross JF (1993) Analysis of oxygen transport to tumor tissue by microvascular networks. *Int J Rad Oncol Biol Phys* **25**: 481–489

Sehgal CM, Arger PH and Pugh CP (1995) Sonographic enhancement of renal cortex by contrast media. *J Ultras Med* **14**: 741–748

Southwick PL, Ernst LA, Tauriello EW, Parker SR, Mujumaar RB, Mujumaar SR, Clark HA and Waggoner AS (1990) Cyanine dye labeling reagents – carboxymethylindocyanine succinimidyl esters. *Cytometry* **11**: 418–430

Stone HB, Brown MJ, Phillips TL and Sutherland RM (1993) Oxygen in human tumors: correlations between methods of measurement and response to therapy. *Radiat Res* **136**: 422–434

Thomlinson RH and Gray LH (1955) The histological structure of some human lung cancers and the possible implications for radiotherapy. *Br J Cancer* **9**: 539–579

Vaupel P, Kallinowski F and Okunieff P (1989) Blood flow, oxygen and nutrient supply, and metabolic microenvironment of human tumors: a review. *Cancer Res* **49**: 6449–6465

Wallen CA, Michaelson SM and Wheeler KT (1980) Evidence for an unconventional radiosensitivity of rat 9L subcutaneous tumors. *Radiat Res* **85**: 529–541

Woods MR, Lord EM and Koch CJ (1996) Prediction of hypoxic radioresistance by monoclonal antibody reactive with 2-nitroimidazole adducts. *Int J Radiat Oncol Biol Phys* **34**: 93–101

© Cancer Research Campaign 1997